# New Antimicrobial Options in the Clinical Practice of Infections Caused by Difficult-to-Treat Pathogens: A Global Opportunity for Public Health

**DOI:** 10.3390/antibiotics11060740

**Published:** 2022-05-31

**Authors:** Luigi Principe

**Affiliations:** Clinical Pathology and Microbiology Unit, “S. Giovanni di Dio” Hospital, 88900 Crotone, Italy; luigi.principe@gmail.com

Antimicrobial resistance (AMR) is a serious cause of concern for public health. Difficult-to-treat pathogens are diffused worldwide, and related infections are associated with significant fatality rates. Recently approved antibiotics represent new weapons in the battle against AMR. However, pan-drug-resistant (PDR) or extensive drug-resistant (XDR) pathogens, mostly belonging to metallo-β-lactamase (MBL)-producing *Enterobacterales*, *Acinetobacter baumannii*, and *Pseudomonas aeruginosa*, remain an important issue for treatment purposes. Moreover, resistance to new antimicrobials is being increasingly reported.

This Special Issue considers all aspects of infections related to difficult-to-treat pathogens and new antimicrobial options. In particular, regarding new antimicrobial alternatives, the paper of Carcione et al. reports the antimicrobial activity of the most promising commercially available antibiotic, cefiderocol, against molecularly characterized *A. baumannii* clinical isolates, most of which were OXA-23 producers [1]. Cefiderocol MICs ranged from 0.5 to 1 mg/L for OXA-23 non-producing strains and from 0.25 to >32 mg/L for OXA-23 producers using the broth microdilution method. Cefiderocol MIC_90_ was 8 mg/L. The diameter of the inhibition zone of cefiderocol ranged from 18 to 25 mm for OXA-23 non-producers and from 15 to 36 mm for OXA-23-like producers using the diffusion disk method. However, a large variability and a low reproducibility were observed during the determination of the diameter inhibition zone, highlighting the important methodological issues for this antibiotic for susceptibility determination. Molecular characterization showed that all isolates presented the ISAba1 genetic element upstream of the *bla*_OXA-51_ gene. *A. baumannii* is usually an XDR pathogen, mostly susceptible only to colistin. This study highlights the potential role of cefiderocol as an anti-*Acinetobacter* antibiotic, showing activity against both carbapenem-susceptible and non-susceptible strains [1].

Another recently approved antibiotic, meropenem/vaborbactam, represents the first antimicrobial option against KPC-producing *Enterobacterales*. Oliva et al. report the first description of a case of septic thrombosis sustained by KPC-producing *K. pneumoniae* unresponsive to ceftazidime/avibactam, which relapsed first with meropenem/vaborbactam monotherapy and was subsequently cured with a combination of meropenem/vaborbactam plus fosfomycin [2]. Septic thrombosis caused by Gram-negative bacilli represents a subtle and often misleading condition that is potentially fatal if not recognized early. This condition often requires prolonged antimicrobial therapy and anticoagulation. This case highlights the possibility of ceftazidime/avibactam underexposure on the infected thrombus and the risk of an in vivo emergence of ceftazidime/avibactam resistance in the setting of persistent bacteremia and sub-optimal anticoagulation. The in vitro study in question demonstrated a high level of meropenem/vaborbactam plus fosfomycin synergism that possibly allowed the definitive resolution of the endovascular infection [2]. Gaibani et al. evaluate the incidence of meropenem/vaborbactam resistance in a KPC-producing *Klebsiella pneumoniae* bloodstream infection in a large Italian hospital [3]. Although rare, meropenem/vaborbactam resistance was found in 8% (*n* = 5) of strains, while 5% (*n* = 3) of strains exhibited cross-resistance to ceftazidime/avibactam. Genomic analysis revealed that meropenem/vaborbactam resistance was associated with truncated OmpK35 and the insertion of glycine and aspartic acid within OmpK36 at position 134–135 (GD134–135). Notably, no specific mutation was associated with cross-resistance. This study indicated that resistance to meropenem/vaborbactam was due, in this instance, to porin mutations, and is associated with reduced susceptibility to both ceftazidime/avibactam and carbapenems [3].

Among new antibiotics, ceftobiprole combines an excellent spectrum for community-acquired pneumonia (CAP) and hospital-acquired pneumonia (HAP) pathogens. The review of Lupia et al. reports the available evidence regarding ceftobiprole use in pneumonia and invasive bacterial infections, shedding light on ceftobiprole stewardship [4]. The clinical application and real-life experiences of using ceftobiprole for bloodstream infections, including infective endocarditis, are limited, but nevertheless promising. In addition, extended-spectrum ceftobiprole activity, including *Enterococcus faecalis*, *Enterobacteriaceae*, and *Pseudomonas aeruginosa*, has theoretical advantages for use as empirical therapy in bacteremia potentially caused by a broad spectrum of microorganisms, such as catheter-related bacteremia [4]. 

In recent decades, stilbenes have aroused great interest because of their high bioavailability, as well as their manifold biological activity. The research efforts of Bongiorno et al. are focused on synthetic heteroaromatic stilbene derivatives, and represent a potential new type of antibiotic with a wide antibacterial spectrum [5]. A preliminary molecular modeling study and a versatile synthetic scheme allow them to define eight heteroaromatic stilbene derivatives with potential antimicrobial activity. Biological assays show excellent bacteriostatic activity for these derivatives, compared to similar molecular structures previously reported, thus paving the way for a new class of antibiotic compound [5].

*Pseudomonas aeruginosa* represents, among the nosocomial pathogens, one of the most serious threats to human health, both for the severity of its clinical manifestations and its ability to develop complex profiles of resistance. Del Giacomo et al. retrospectively collected the data of 21 patients admitted to a tertiary-care University Hospital of Rome with infections due to XDR *P. aeruginosa* isolates during the second half of 2020 [6]. Rates of resistance to ceftolozane/tazobactam and ceftazidime/avibactam in these XDR strains were consistent, while susceptibility to colistin was preserved in all the tested samples. The authors conclude that caution is needed when new antibiotics are administered. In some cases, an old antibiotic such as colistin, even with its known safety and efficacy limits, can still represent the only available therapeutic option [6]. 

Metallo-β-lactamases (MBLs) are among the most challenging bacterial enzymes to overcome. Among old antibiotics, aztreonam is the only β-lactam not hydrolyzed by MBLs; instead, it is often inactivated by co-produced extended-spectrum β-lactamases (ESBLs). From a therapeutic point of view, MBL producers still remain an unsolved problem. In their study, Morroni et al. assess the activity of combinations of aztreonam with old and new β-lactamases inhibitors (BLIs) against MBLs and ESBLs co-producing Gram-negative clinical isolates [7]. Aztreonam plus avibactam was found to be the most effective combination. In addition, relebactam and zidebactam showed to be effective, but with smaller reduction in the MIC of aztreonam. However, no effective combination was found for some non-fermentative bacilli (i.e., *Elizabethkingia meningoseptica* and *Chryseobacterium indologenes*), suggesting the presence of additional resistance mechanisms that complicate the choice of an active therapy [7]. 

The application of molecular diagnostics and whole genome sequencing represents an important approach to better understand antimicrobial resistance mechanisms. In particular, De Angelis et al. show the performance of T2Resistance™, which allows the detection of carbapenemase- and other β-lactamase-encoding genes directly from blood samples [8]. The rapid detection of important resistance genes provides significant benefits related to the reduction in empirical therapy, to antimicrobial stewardship, and to hospital infection control. Piazza et al. describe the dynamics of the largest Italian hospital outbreak sustained by KPC-producing *Escherichia coli* by WGS, involving 106 patients and 25 hospital wards, during a six-month period [9]. KPC-producing *E. coli* remains uncommon, being mainly reported as the cause of sporadic episodes of infection rather than outbreak events. In this study, the isolates were described in detail at both genomic and phylogenomic levels in order to trace their spread among the hospital wards. In particular, the outbreak was sustained by the pandemic clone ST131. This KPC-producing clone originated in a previous KPC-producing *K. pneumoniae* endemic context probably by plasmid transfer. Transmission of the *bla*_KPC_ gene to the globally disseminated high-risk ST131 clone represents a serious cause of concern. The application of WGS in outbreak investigations could be useful to better understand the evolution of epidemic events in order to address infection control and contrast interventions, especially when high-risk epidemic clones are involved [9].

Additionally, pathogens with a fully susceptible profile, but with important virulence factors, could be a serious cause of concern. This is the case of the hypervirulent *K. pneumoniae* (hvKp). Although often multi-susceptible to antibiotics, hvKp variants can cause severe infections, including hepatic abscesses, bacteremia, and meningitis, with a particularly disconcerting propensity to cause community-acquired, life-threatening infection among young and otherwise healthy individuals. Piazza et al. report the clinical characteristics of a hypermucoviscous *K. pneumoniae* (hmKp) strain isolated in Italy that instigated sustained and recurrent meningitis in a patient of Peruvian origin [10]. The strain was susceptible to most antibiotics. WGS detected some resistance genes, including *bla*_SHV-11_, *bla*_SHV-67_, *fosA*, and *acrR*. The isolate belonged to ST65 and serotype K2, and exhibited several virulence factors related to the hvKp variant. Among these were the siderophore genes entB, irp2, iroN, iroB, and iucA, the capsule-regulating genes rmpA and rmpA2, and the type 1 and 3 fimbriae fimH27 and mrkD. Fortunately, these strains are commonly associated with antibiotic multi-susceptible profiles, but resistant variants are emerging. The rapid detection of hmKp/hvKp phenotypes, together with optimal antibiotic treatment, could be of utmost importance for the clinical resolution of severe invasive infections sustained by these strains [10].

Infections sustained by MDR, XDR or hypervirulent pathogens are still diffused worldwide. New antimicrobials and new diagnostic techniques are now commercially available; however, they are not enough. To limit this trend, the application of both antimicrobial and diagnostic stewardship concepts is urgently needed in future practice.
